# Dengue, West Nile, and Zika Viruses: Potential Novel Antiviral Biologics Drugs Currently at Discovery and Preclinical Development Stages

**DOI:** 10.3390/pharmaceutics14112535

**Published:** 2022-11-20

**Authors:** Ivo C. Martins, Rafaela C. Ricardo, Nuno C. Santos

**Affiliations:** Instituto de Medicina Molecular, Faculdade de Medicina, Universidade de Lisboa, Av. Prof. Egas Moniz, 1649-028 Lisbon, Portugal

**Keywords:** flavivirus, dengue virus, West Nile virus, Zika virus, therapeutics

## Abstract

Dengue, West Nile and Zika viruses are vector-borne flaviviruses responsible for numerous disease outbreaks in both Hemispheres. Despite relatively low mortality, infection may lead to potentially severe situations such as (depending on the virus): hypovolemic shock, encephalitis, acute flaccid paralysis, Guillain-Barré syndrome, congenital malformations (e.g., microcephaly) and, in some situations, death. Moreover, outbreaks also have major socioeconomic repercussions, especially in already vulnerable societies. Thus far, only generic symptoms relief is possible, as there are no specific treatments available yet. Dengvaxia was the world’s first dengue vaccine. However, it is not fully effective. Prophylactic approaches against West Nile and Zika viruses are even more limited. Therefore, therapeutic strategies are required and will be discussed hereafter. We will first briefly present these viruses’ epidemiology, life cycle and structure. Then, we introduce the clinical presentation, diagnosis approaches and available vaccines. Finally, we list and discuss promising compounds at discovery and preclinical development stages already deposited at the GlobalData database and divided into three main types, according to therapeutic molecule: antibody-based, peptide-based molecules and, other compounds. To conclude, we discuss and compare promising developments, useful for future therapies against these three flaviviruses of major concern to human health.

## 1. Introduction

Flaviviruses are single-stranded RNA viruses [1], being one of the most clinically relevant virus group amongst arboviruses [2]. Viruses of the *Flaviviridae* family are responsible for a spectrum of human diseases ranging from mild self-limited illness to severe life-threatening syndromes [1]. Several of these viruses can easily adapt to different hosts and environmental conditions, making them an epidemiological challenge that is somewhat difficult to manage and contain [3]. The global widespread and epidemic transmission over the last seven decades of several members of the *Flavivirus* genus, namely dengue (DENV), West Nile (WNV) and Zika (ZIKV) viruses, has been noteworthy [1]. Increasing unplanned urbanization, which tends to create ideal arthropod breeding habitats, extensive global travel and international trade (facilitating virus and vector geographical spread), environmental changes (namely climate change), and biological challenges (inherent to viral vectors management) are some of the factors that contributed to these viruses’ expansion [1,4].

This is clear concerning dengue as, since the turn of the millennium, the scientific community witnessed an increase in its incidence. According to the World Health Organization (WHO), about half of the world’s population may be at risk of DENV infection [5]. Besides the worrisome impacts on the populations’ health, dengue infections also have repercussions on the affected regions’ economy. The estimated total annual aggregate economic burden of dengue reached 8.9 billion USD in 2013, showing the problem dimension [2].

Regarding WNV, infections are also associated with economic losses, not only related to treatment costs and morbidity losses, but also with intensive preventive control programs, plus the loss of animals/animal products [6]. Historically, WNV outbreaks causing febrile illness occurred sporadically in regions of Africa, the Middle East, Asia and Australia. Notwithstanding, in the 1990s, cases in Eastern Europe were associated with neurological disease and deaths [1] and, more recently, outbreaks have been reported in non-endemic regions [6].

**Figure 1:**
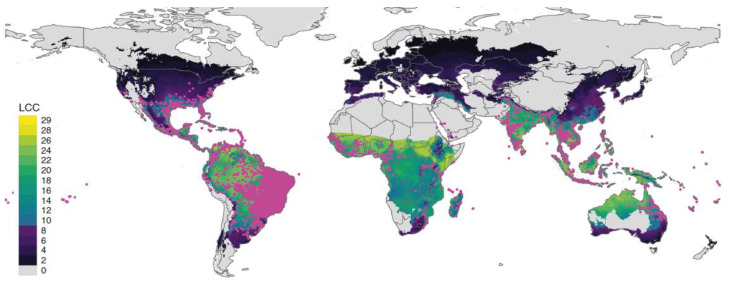
*Aedes aegypti* distribution worldwide. The map indicates the total number of annual life-cycle completions (LCC) of *A. aegypti*, with occurrence data overlaid. *A. aegypti* is a recognized competent vector for both DENV and ZIKV. Adapted with permission from Ref. [12]. Copyright 2020 Iwamura, T.; Guzman-Holst, A.; Murray, K.A.

Concerning ZIKV, this flavivirus shares the same main vectors as DENV, namely *Aedes albopictus* and *Aedes aegypti* mosquitoes [4]. During the 2015–2016 ZIKV epidemic outbreak in Brazil, an association between ZIKV infection and microcephaly in newborns was reported [4]. One year later, the WHO declared ZIKV infection as a Public Health Emergency of International Concern. Other outbreaks soon appeared and, thus far, a total of 86 countries and territories have disclosed to the WHO evidence of mosquito-transmitted ZIKV infection [7]. As *Aedes* spp. mosquitoes are further expanding (Figure 1), so are the risks of future infections in areas of the planet where these diseases are not expected.

As previously mentioned for WNV infection, several control and prevention strategies aimed at vector control have also been implemented for DENV and ZIKV. These strategies encompass mechanical, chemical and biological methods, including methods such as surveillance through geographical mapping of virus foci, oviposition-based techniques, use of insecticides and plant derivatives, bacterial infection of vectors (e.g., Wolbachia, a parasite that interferes with essential mechanisms of the vector species) and genetic manipulation of mosquitoes [8,9,10]. In any case, direct measures against the mosquito vectors have been the most effective. These include simple approaches, such as disposal of containers serving as stagnant water deposits, which are easy to implement and constitute one of the most reliable strategies to avoid vector proliferation [9]. Other measures specifically aimed at ZIKV infection are also recommended, such as safe sexual practice (given the possibility of sexual transmission) and avoiding travelling to endemic regions during pregnancy [9,11]. Community-based control programs that promote the education of at-risk populations are also important [8].

Overall, as the vectors responsible for infection spread to other than tropical and subtropical regions, the diseases they convey are becoming more acknowledged by health services [4]. Therefore, the continued threat posed by flaviviruses highlights the imperative need for prophylactic approaches, as well as effective treatments, to alleviate their major health impact and financial burden in affected regions [1,4].

## 2. Epidemiology

**Figure 2:**
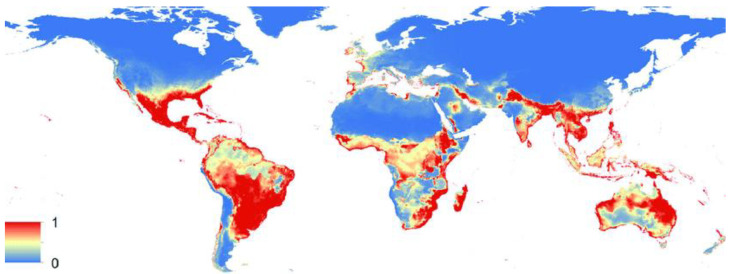
Estimated potential global distribution of *Culex quinquefasciatus*. The colors represent the suitability level from 0 (blue) to 1 (red). *C. quinquefasciatus* is a known competent biological vector of WNV. In addition, recent evidence suggested its potential as vector for ZIKV [13,14]. Adapted with permission from Ref. [13]. Copyright 2018 Alaniz, A.J.; Carvajal, M.A.; Bacigalupo, A.; Cattan, P.E.

In recent decades, we have witnessed the emergence and re-emergence of dengue, Zika and West Nile viruses in both the Northern and Southern Hemispheres. DENV and ZIKV are now two of the most epidemiologically concerning viruses globally [2]. Hereafter, the epidemiologic aspects surrounding these viruses (and which help to explain their global prominence) will be discussed, to understand the key issues to be considered. One key factor explaining these viruses global spread is the concomitant expansion of their vectors, as exemplified here for *A. aegypti* and *Culex quinquefasciatus* mosquitoes, which global distribution has been spreading (Figure 1 and Figure 2, respectively).

### 2.1. Dengue Virus

Precise determination of DENV incidence is challenging, as most cases are asymptomatic or mild, adding to that the underreporting of cases due to misdiagnosis as other febrile illnesses. However, estimates of the number of annual infections worldwide range from 284 to 528 million [2]. A report including 76 countries indicated that, between 1990 and 2013, apparent cases of dengue more than doubled every decade [2], with the number of cases reported to WHO in the last two decades increasing over 8-fold, reaching 5.2 million in 2019 [5]. DENV origins are thought to remount to non-human primates (sylvatic DENV) in Africa and Asia, estimated to have emerged 1000 years ago. Cross-species transfer to humans then occurred independently for all four serotypes (DENV1 to DENV4), and transmission in human populations has been established in the last few hundred years [2]. Nowadays, DENV is endemic in many regions of Africa, the Americas, Eastern Mediterranean, Southeast Asia and Western Pacific. The highest incidence rates occur in Southeast Asia, with an age-standardized yearly average of 34.3 cases per 1000 inhabitants [2]. In fact, the WHO stated that Asia represents about 70% of the current burden of disease globally [5]. Studies suggest that silent infections play a substantial role during dengue epidemics and may contribute up to 84% of total DENV transmission [15]. In the last few years, outbreaks have also been reported in Europe, namely autochthonous dengue cases in Croatia and France in 2010 [4]. Later, in 2012–2013, 1080 dengue cases were confirmed in Madeira Island, Portugal, the largest European outbreak since 1928, when more than one million people were affected in Greece and Turkey [4].

### 2.2. West Nile Virus

WNV was first isolated in 1937, from a febrile patient in Uganda, West Nile Province [1,6,16,17]. Early epidemics studies associated WNV with relatively mild disease in humans. Serosurveys also suggest that WNV outbreaks may have by then occurred throughout much of Africa, the Middle East and South Asia, albeit no clear evidence of clinical cases is available [16]. The most prominent WNV outbreaks with clinical relevance have taken place in Israel, Romania, Russia, Greece, and the USA [18]. In Israel, WNV was first isolated from a febrile child in 1951, during an outbreak near the Israeli city of Haifa, 14 years after the first identified case, in 1937, in the former West Nile district of Uganda [16]. Later, in 1957, the first deaths due to WNV neuroinvasive disease (WNND) were reported in elderly Israeli patients. In 2000, 417 confirmed cases and 35 deaths were attributable to WNV and, ever since, Israel suffers summertime outbreaks of varying severity [16]. In Europe, the first WNV cases occurred at Albania, in 1958 [16]. In 1962–1963, the first European WNV outbreak occurred, in Southern France, causing both human and equine disease [16]. Since then, Europe endured two large WNV epidemics [16,18]. The first took place in Romania, in 1996, with 17 deaths registered, and the second occurred in Russia, in 1999, with 40 deaths from acute aseptic meningoencephalitis consequent to WNV infection [16]. Several other outbreaks and occasional cases have impacted European countries, and, as such, WNV surveillance programs are now implemented in some countries [16]. More recently, in 2010, in Northern Greece (between the rivers Axios and Aliakmonas) an outbreak resulted in a total of 262 patients, 65 of which classified as West Nile fever, while 197 suffered neurological disease [17]. The virus has also been isolated from mosquitoes in Portugal and the Czech Republic, migrating birds in Slovakia and Western Ukraine, and ticks in Hungary and Moldavia [16]. In the USA, a well-known WNV outbreak occurred in the summer of 1999, in New York [1,16,19], in a cluster of encephalitis patients [16]. In the following years, the virus spread to all 48 contiguous USA states, into Canada, Mexico [16], the Caribbean and even part of South America [1]. WNV is now endemic in the USA, causing 3 of the largest arboviral neuroinvasive disease outbreaks in the country’s history [19]. WHO now considers WNV endemic in Africa, the Middle East, the USA, Australia, Europe and Asia [18], demonstrating the virus ability to successfully propagate around the globe.

### 2.3. Zika Virus

Zika virus was first identified by chance in 1947 in a rhesus monkey of the Zika Forest, Uganda, amidst studies to discover the vector responsible for the transmission of the yellow fever virus [4,14,20,21,22]. Sometime later, the first cases of human infection were reported in Uganda, Tanzania and Eastern Nigeria [4,21,22]. In the following years, scarce, geographically limited cases were reported, mostly describing patients with clinical presentations consistent with mild febrile illnesses [1,4]. Surveillance studies described possible human infections occurring throughout Africa, Asia and Oceania [1,21], although some authors consider that results may overestimate true prevalence of the virus, as serologic overlap often occurs between ZIKV and other flaviviruses (including DENV and WNV) [21]. The first major outbreak of human ZIKV infection was reported in 2007 in the Yap islands (Federated States of Micronesia) [4,14,21]. Estimates suggest that approximately 73% of the population was infected; however, only a relatively small number of infected individuals (≈18%) ended up developing symptomatic disease [21]. Since 2007, outbreaks have been reported in various regions of Asia and the Pacific, including French Polynesia, Cook Islands, Easter Island, New Caledonia, Singapore, Vietnam and Thailand [4,14,21]. In 2015, ZIKV infections emerged in continental South America, in Brazil, this time being correlated with the possible occurrence of severe neurological complications, both in adults and infants [1,4,21]. In the same year, Cape Verde also reported an outbreak [4,14]. In Europe, there are records of a small number of imported cases, either travel-associated or cases of sexual and vertical transmission [4,14,21]. To date, we have knowledge of at least 86 countries and territories with reported evidence of ZIKV infection due to mosquito-mediated transmission [7], and Zika virus has been declared a public health emergency [4,21,22].

### 2.4. A Note on Vectors Expansion Possibilities

Part of the difficulty in dealing with DENV, ZIKV and WNV revolves around the characteristics of their insect vectors. Their ability to rapidly expand and establish novel mosquito populations in previously non-endemic areas (as exemplified in Figure 1) increases the probability of new and more frequent outbreaks [4]. Factors promoting viral amplification and human outbreaks are complex and depend on specific vector species. *A. albopictus* and *A. aegypti* mosquitoes are the most effective DENV and ZIKV vectors [1,2,4,13,14]. *Culex* spp. mosquitoes are the main WNV vectors [1,6,19,23], although other mosquito species (e.g., *A. albopictus*) may possess transmission competency [6,16]. Expansion of these vectors is not only dependent on environmental changes, but also on enhanced globalization and socioeconomic factors [4]. Regarding environmental changes, factors such as higher temperature and higher humidity are known to benefit mosquitoes’ populations [23]. Elevated temperatures shorten the incubation time in mosquitoes and increase viral transmission efficiency to hosts [19,23]. However, *A. albopictus* mosquitoes have shown to be able to survive in more temperate regions, a particularity that potentially promotes their expansion to other than tropical and sub-tropical regions [4]. Rapid travel and trade, associated with globalization, allow diseases and their associated vectors to overcome geographic barriers and promote their spread from endemic to non-endemic regions [4]. As previously mentioned, socioeconomic factors have also been associated with higher incidence of flaviviruses’ infections in some locations [1,2,19]. In addition to the already mentioned factors, WNV cycles in nature between *Culex* mosquitoes and vertebrate animal hosts, namely birds, horses and other mammals [1,6,19]. These hosts represent important reservoirs and are essential for the sustainability of the infection cycle, acting as virus amplifiers and source of infection for dead-end-hosts, like humans [1,6]. In fact, the role of migratory birds in WNV introduction and spread across Europe and the Americas has already been recognized [23].

## 3. Symptoms, Diagnosis and Vaccines

**Figure 3:**
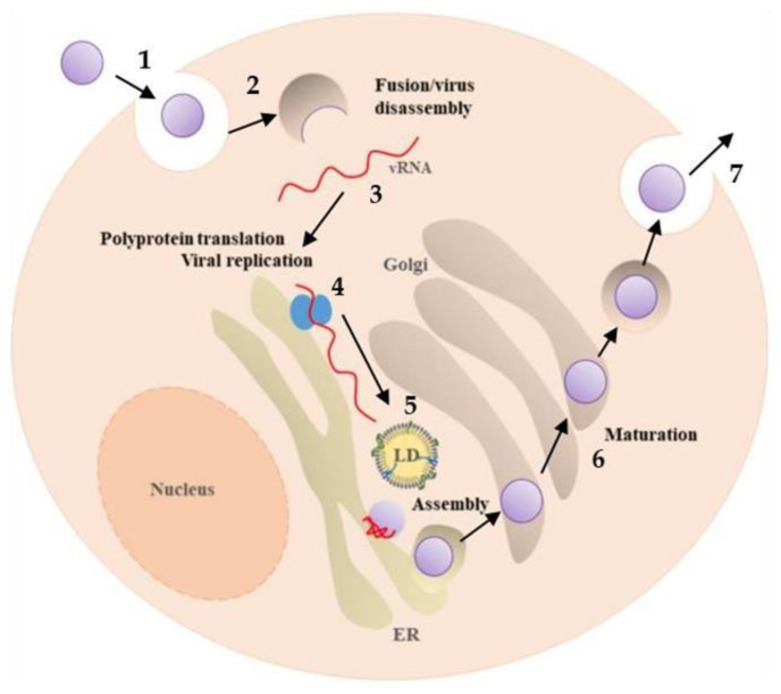
Viral life cycle. After entering host cells by clathrin-mediated endocytosis (1), membrane fusion of the viral envelope and the cell membrane occurs (2). Viral genome is released into the cytoplasm (3) and translated into a single polyprotein, later cleaved into the three structural and seven non-structural proteins (4). Next, replication occurs surrounding the endoplasmic reticulum (ER) and lipid droplets (LDs) (5), followed by viral packaging and assembly to form infectious viral particles (6), which are then released through exocytosis (7). The three flaviviruses discussed here share similar virion structure and mode of infection, besides being also all mosquito-borne. Adapted with permission from Ref. [4]. Copyright 2020 Silva, N.M.; Santos, N.C.; Martins, I.C.

Before proceeding, it is important to shortly elaborate on these viruses’ life cycle and structure (Figure 3), which highlights their common origin and partially explains their similar mode of transmission and infection. Briefly, flaviviruses are small spherical viruses of approximately 50 nm in diameter, with a single positive-strand RNA genome, encoding three structural viral proteins—capsid (C), pre-membrane (prM), which is a precursor to membrane (M), and envelope (E)—and seven non-structural viral proteins (NS1, NS2A, NS2B, NS3, NS4A, NS4B, and NS5) [1,24]. The three structural proteins constitute the virus particle, wherein C protein encapsidates the ~10.8-kb genome and is surrounded by a host-derived lipid bilayer incorporating copies of the E and M proteins [24].

Flaviviruses’ life cycle includes as main steps the viral binding and entry, translation, replication, assembly, and release [25]. The entry process begins with the attachment of viral particles to the cell surface and binding of the viral E protein to a cellular receptor [1,25]. Identifying the specific entry receptor involved in the internalization of infectious virions in humans and other vertebrate animals remains a challenge [1,25], but mannose and phosphatidylserine receptors have been reported as relevant for flavivirus pathogenesis [2,25]. Several cell surface markers have also been proposed as attachment factors, such as glycosaminoglycans, C-type lectins DC-SIGN (dendritic cell-specific intracellular adhesion molecule-3-grabbing non-integrin), heat-shock proteins, the chaperone BiP/GRP78, neolactotetraosylceramide and CD14 [2,25].

After attachment, clathrin-dependent endocytic vesicles mediate virus internalization and membrane fusion is triggered by the endosomal acidic environment [1,2,4,25]. Upon fusion of the viral envelope and cell membrane, the RNA genome is released into the cytoplasm and translation of the viral polyprotein it encodes occurs [2,4]. This is followed by the cleavage by host and viral proteases into the structural and non-structural proteins [2,4,25].

Flaviviruses assembly and replicate on the endoplasmic reticulum (ER) membranes [26]. Immature virions are transported through the secretory pathway [2] and maturation is then promoted by the acidic pH of the trans-Golgi network [27]. It is at this stage that prM is cleaved into M by a host-encoded furin protease, causing the spiky virus surface (characteristic of immature virions) to transform into a smoother surface with the typical morphology of mature virions [2]. Finally, mature virions are released from host cells through exocytosis [2,4].

Concerning viral proteins functions, the C protein has key roles in viral assembly, genome encapsidation and interaction with host lipid systems [2,28,29]. The prM protein interacts with the E protein, preventing conformational changes that could allow fortuitous fusion of virions with host membranes during egress, and its cleavage to M is required for formation of mature virions [1]. The E protein is one of the most important for binding. This protein contains epitopes that bind cell receptors, enabling target recognition and viral entry [30]. The seven non-structural proteins are necessary for effective viral replication [30]. Thus, these viruses display an overall common structural arrangement of the virion structure and a very similar proteome. However, and notwithstanding some symptoms that can be common among them, this shared structural resemblance does not imply similar clinical features, as discussed in the next section.

### 3.1. Symptoms and Clinical Presentation

Despite these viruses’ similarity at the virion structure level, mode of infection and terms of transmission, symptoms can be quite different, both between viruses (WNV vs. ZIKV vs. DENV vs. each of these viruses’ serotypes/strains) and between infected people. Acute flavivirus infection in humans span conditions ranging from asymptomatic to mild illness and up to severe, even fatal, disease [1], as described in Table 1. Estimates vary widely, but, roughly, 40 to 80% of flavivirus infections are considered asymptomatic or to cause minimal illness [1,4,14,16,19,21,23]. Estimations are difficult in part because these viruses’ infection can be initially confused with common flu (frequently leading to misdiagnosis and under-reporting of flaviviruses infections). The infection only becomes more easily identifiable if it evolves into a potentially life-threatening clinical presentation, with more specific symptoms, as briefly described ahead.

#### 3.1.1. DENV Infection

Concerning symptomatic DENV infection, typical clinical presentation consists of a self-limited flu-like syndrome (Table 1), with patients experiencing fever, headache, myalgia, arthralgia and sometimes developing rash [1]. Such symptomatic dengue infection normally comprises three stages: the febrile, critical and recovery phases [2,30]. The febrile phase is characterized by a sudden fever onset, often accompanied by malaise, vomiting, constitutional symptoms, and the previously mentioned symptoms [30]. The critical period begins at the time of defervescence [2,30]. Individuals require close monitoring to promptly identify possible signs of vasculopathy, namely increased vascular permeability, plasma leakage, and intravascular volume depletion [4,30]. Identifiable signs include increased hemoconcentration, serosal effusions, most frequently pleural and peritoneal, and gall bladder wall oedema. Minor hemorrhagic complications may also be seen during this critical phase [1,4,30]. Dengue shock syndrome is evident when pulse pressure values reach 20 mmHg or lower, and requires rapid fluid resuscitation [30]. Other complications, resulting from organ impairment, have also been documented, but most likely ensue in individuals with underlying conditions. According to some authors, recurrent episodes of shock can occur in the 48–78 h interval before resolution of the vasculopathy and are associated with increase in fatal outcomes. Following appropriate supportive care, full recovery typically happens within 1–2 weeks. However, sequalae such as fatigue, weakness, myalgia and depression may last up to several months after acute disease resolution in adult patients [30]. According to the 2009 WHO dengue case classification, severe dengue occurs when symptomatic individuals experience at least one complication related to plasma leakage, and that originates dengue shock syndrome or respiratory distress, severe hemorrhage or organ impairment [30]. Overall, less than 5% of DENV infections progress to the life-threatening severe dengue clinical presentation [4].

#### 3.1.2. WNV Infection

WNV infection cases are mostly asymptomatic, being estimated that less than 1% of infected individuals progress to severe disease [16,23,31]. Severe West Nile disease most commonly manifests as neuroinvasive conditions comprising (Table 1): West Nile meningitis, West Nile encephalitis, and acute flaccid paralysis [23,31]. The clinical presentation of West Nile meningitis resembles those caused by other etiological agents. Individuals presenting fever, headache, neck stiffness, nuchal rigidity, photophobia, and Kerning’s and Brudzinski’s signs reflecting meningeal irritation can be positive upon physical examination [19,31]. Patients developing West Nile encephalitis may present an altered level of conscience [6,31], and focal neurological signs and symptoms, such as dysarthria [31], tremor, ataxia [19,31], and parkinsonism [19,31]. Albeit most West Nile fever patients have complete recovery, those with neuroinvasive disease have poorer outcomes [16,23,31]. Recent studies suggest that, within this patients’ cluster, individuals with West Nile encephalitis had worse outcomes and required more assistance after hospitalization than patients who develop West Nile meningitis [31]. As previously mentioned, acute flaccid paralysis may also develop, most frequently as an acute asymmetric paralysis with normal sensory examination [31]. One study documented that most patients did not have viral prodrome or signs of meningitis or encephalitis before flaccid paralysis onset [31]. The same authors followed a group of paralysis patients and concluded that initial disease severity was not predictive of outcome [31]. Other studies indicate that neuroinvasive disease recovery time is highly variable, with physical and cognitive deficits persisting from 6 months to 2 years after initial diagnosis [16,23,31]. Among other risk factors of severe West Nile disease, immunosuppression and old age seem to be the most important [16,23]. This is crucial information, relevant in epidemiological terms and monitoring, both at the population as well as at the individual level. The aging of the population (alongside the known and well-documented vector and virus worldwide global expansion) should thus be computed, namely when considering resources allocation to R&D, monitorization and public health WNV policies.

#### 3.1.3. ZIKV Infection

Regarding ZIKV infections, only a small percentage seems to result in complicated clinical outcomes [4]. Different flaviviruses are known to have different cellular and tissue tropism. ZIKV can cause both visceral and neurotropic disease, preferentially infecting progenitor cells, epithelium and myeloid cells, and produces injury on the reproductive tracts and eyes [1]. ZIKV has also tropism for placental tissue, which may explain its teratogenicity [1]. As shown in Table 1, ZIKV infection has been associated with cases of microcephaly and other congenital malformations [4]. In adults, severe neurologic complications of infection described include Guillain- Barré syndrome [1,4,21], but also meningitis and meningoencephalitis [21]. Three ZIKV lineages have been identified but, despite being now clear that African lineage strains are more virulent than Asian ones, it is still not known whether increased virulence of certain strains may result in more severe clinical outcomes [4]. Overall, ZIKV monitoring is necessary to collect more data but, given the consequences to newborns and their families, this virus must also be considered in public health monitoring policies. Moreover, as *Aedes* spp. are also found throughout the globe, ZIKV incidence is only expected to increase, as already seen for DENV, which is transmitted by the same vectors.

### 3.2. Diagnosis

Diagnosis of these flaviviruses’ infections is complicated, due to the wide range of possible clinical presentations (described above). Moreover, the reason why some infected people develop more severe disease phenotypes than other is also still not fully understood, making it more difficult to reach proper diagnosis and prognosis. Host factors, including polymorphisms in key host genes, prior flavivirus immunity (primary vs. secondary infection), host immune status, age and the presence of certain comorbidities, such as hypertension and diabetes, have been suggested as predisposing to severe disease [1,2,4]. Moreover, the specific tropism of each virus, the ability of evading host immunity and direct pathogenic effects are also mentioned as viral factors that likely contribute to the variability in pathogenicity amongst viral strains [1]. Ideally, all these factors should be considered for a proper diagnosis.

When DENV infection is suspected, the choice of diagnostic test depends on the time elapsed since disease onset [2,30]. In the first 5 days, dengue may be diagnosed by virus isolation in cell culture, detection of viral RNA by nucleic acid amplification tests (NAAT) such as reverse transcription polymerase chain reaction (RT-PCR), or detection of viral antigens such as NS1 by enzyme-linked immunosorbent assay (ELISA) or rapid tests. After this period, specific IgM or IgG antibody detection through serological assays should be preferred, as viruses subside and dengue-specific antibodies begin to appear [2,30]. Dengue IgM antibodies may persist until 3 months after secondary infection or longer in primary dengue infections. At point of care, combination of NS1 antigen detection and IgM testing offers a longer diagnostic period, although cross-reactivity with ZIKV has been reported for both [21,30].

Pertaining to ZIKV infection diagnosis, the same rationale applies. Studies estimated that ZIKV viremic period may be as brief as 5 days, and during this period, molecular amplification using RT-PCR on serum samples seems to be the most specific diagnostic method [21]. Serologic approaches have limitations, as cross-reactivity with DENV is likely to occur, as previously mentioned. Currently, serum or cerebrospinal fluid are the samples of choice for testing; however, the utility of other specimens, such as urine, are being evaluated, and according to one study, ZIKV RNA may be detectable up to 20 days after viremia becomes imperceptible [21].

For WNV neuroinvasive diseases, a definite diagnosis requires a positive IgM antibody test in the serum or cerebrospinal fluid, when clinical presentation is suggestive of either one of the three known syndromes (meningitis, encephalitis and acute flaccid paralysis) [31]. The diagnosis should be considered when epidemiological data suggests a likely context, as in endemic regions during seasons when mosquito-borne diseases tend to occur [6,31]. For differential diagnosis with flaviviruses of the Japanese and thick-borne encephalitis complex, serological assays and PCR testing are helpful, as clinical presentations do not differ [23,31]. Additional acute flaccid paralysis differential diagnosis includes conditions such as Guillain-Barré syndrome, myopathy, neuromuscular junction disorders and other motor neuron diseases caused by alternative viral agents. In this case, electrophysiological and neuroimaging studies may provide helpful clues for the distinction between possible etiologies [31].

### 3.3. Vaccines

Before proceeding towards therapeutic possibilities, it is relevant to include a short description on prophylactics, namely available vaccines. The world’s first dengue vaccine, CYD-TDV or Dengvaxia, is a live attenuated, tetravalent vaccine, based on the YFV-17D vaccine backbone, developed by Sanofi Pasteur [1,30]. CYD-TDV performance efficacy depends on serotype, baseline serostatus and age [4,30]. A large phase III clinical trial in Asia and Latin America revealed an increment in risk of severe dengue in seronegative vaccine recipients in relation to seropositive recipients not previously vaccinated [30]. In 2018, the WHO stated that pre-vaccination screening should be performed in countries considering CYD-TDV vaccination [30]. CYD-TDV is currently approved in several countries, with indication for individuals aged 9 to 45 years old, who had at least one previous DENV infection [1,4,30]. Although a final verdict is still to be issued, a mounting body of evidence indicates that the dengue vaccine Dengvaxia can promote the formation of cross-reactive antibodies that may have a role in triggering antibody-dependent enhancement (ADE) of flavivirus infections subsequent to the vaccination in otherwise seronegative patients [32,33,34,35]. Nevertheless, other researchers suggest that ADE due to vaccination is a rare phenomenon, if it occurs at all [36]. Further research is necessary to clarify these aspects.

As for WNV and ZIKV human infection, there are currently no approved human vaccines or other specific treatments available [1,4,31]. Even though progress towards development of potential WNV vaccines has been made, their cost-effectiveness for human treatment remains uncertain [23]. Notwithstanding, a WNV vaccine for equine use has been approved. It is based on immunization with formalin-inactivated WNV, a recombinant canarypox virus vector, and a DNA plasmid expressing WNV prM and E proteins [31]. Considering all of this, developing effective therapeutic approaches remains a major need, even if more prophylactic vaccination strategies become available, as, so far, even in the case of DENV, no vaccine is either fully efficient against all viral serotypes, or recommended and effective in all age groups, namely the most vulnerable. Thus, the most promising advances in terms of possible therapeutic approaches are described hereafter.

## 4. Potential Novel Drugs for Flavivirus Infections Treatment

Given the above, it is important to determine the most promising biomedical advances in terms of future treatments against these flaviviruses. To do so, we accessed the GlobalData database on 22 April 2021 (www.globaldata.com) and searched for treatments disclosed as being currently developed against flavivirus infections. Selected data included molecules at discovery and preclinical development stage consisting of peptides, oligonucleotides, and proteins targeting flaviviruses. As keywords for targets, we included Flavivirus, as well as the particular virus mentioned (search terms: flavivirus, dengue, West Nile, Zika, DENV, WNV, ZIKV). This yielded 10 relevant hits, which were further studied and classified. These include, when classified at the molecular level, antibody-based, peptide-based and other approaches, as described ahead.

### 4.1. Antibody-Based Therapeutic Approaches

Hereafter, five antibody based-therapeutic approaches are presented. All have been evaluated in detail, being in different stages of the clinical development process. In the concluding remarks section, a comment on those showing the most promising advances is available.

#### 4.1.1. AC-10

In their studies, Bailey et al. characterized several neutralizing monoclonal antibodies (mAbs) isolated from a patient with acute Zika virus infection [37]. Their purpose was to map the epitopes targeted by neutralizing antibodies and try to understand whether certain germline rearrangements provided better neutralizing responses [37]. AC-10 was one of four antibodies that demonstrated high neutralizing potency against ZIKV [37]. The rearrangement of VH1-2/VL2-8 (with VH and VL referring to heavy and light variable regions, respectively) was a common ground in potently neutralizing antibodies, including AC-10, as well as the presence of a motif composed of at least three tyrosine residues in the complementary-determining region 3 [37]. AC-10 was shown to be potently neutralizing, with 50% inhibitory concentration (IC_50_) values below 25 ng/mL, most likely inhibiting viral binding and/or fusion, as its neutralization efficiency was superior when added before or at the time of infection [37]. AC-10 and other potently neutralizing antibodies induce escape mutations in the lateral ridge region of domains III and I of ZIKV E protein [37]. On the other hand, less neutralizing antibodies tended to induce escape mutations in E protein domain II [37], suggesting that this domain may be less determinant for antibody-mediated protection. Interestingly, point mutations in site 162 of domain I and in site 368 of domain III were detected in escape variants to AC-10, suggesting that these positions may play a more important role in neutralization [37]. Authors concluded that residue S368 in the E protein lateral region was required for complete inhibition by AC-10 and other mAbs containing the same germ line rearrangements [37]. This is an important discovery, as this region is conserved in 97.6% of the ZIKV sequences analyzed [37]. Therefore, AC-10 may be widely effective against different ZIKV strains. In addition, the S368R mutation correlated with the appearance of another mutation in the viral prM gene (D57N), suggesting that prM residue 57 may be key to viral replication [37]. Besides, other mutations were detected in regions encoding nonstructural proteins, namely NS2A, NS3 and NS5 [37]. However, the significance of these mutations is still not fully understood [37]. Regarding Fc-mediated functions, low concentrations of neutralizing antibodies increased ZIKV virions internalization, but did not lead to ADE or any form of antibody-dependent cellular cytotoxicity on infected cells [37]. E protein-specific neutralizing antibodies also did not elicit protective Fc-mediated effector functions [37].

#### 4.1.2. EDE

Another study, by Barba-Spaeth et al., explored neutralizing antibodies against DENV serotypes 1 to 4, which also targeted a quaternary site at ZIKV E protein exposed surface [38]. Antibodies were first isolated from a dengue patient [38]. Crystal structure analysis of these antibodies complexed with ZIKV E protein situated its epitope in the interface between the two subunits of the E protein dimer, at a location believed to be the interaction site of prM with E dimers during virus replication [38]. Two subsets of E-dimer epitope (EDE) antibodies were identified, EDE1 and EDE2, which display a differential requirement for glycosylation on the variable 150 loop of E protein: EDE2 affinity required glycosylation, while EDE1 did not [38]. Neutralization assays suggested that EDE1 antibodies neutralize ZIKV more potently than EDE2 antibodies. EDE1 antibodies neutralized ZIKV African strain HD78788, as well as the French Polynesia PF13 strain (in this case, not showing glycosylation), with IC_50_ values in the nanomolar range [38]. EDE2 binding capacity increases with glycan present; however, EDE2 antibodies can equally neutralize both strains [38]. Most antibodies initially isolated from dengue patients targeted the fusion loop epitope (FLE), contrarily to EDE1 and EDE2 antibodies. Thus, anti-EDE may be appropriate for epitope-focused vaccine against ZIKV/DENV viruses’ serogroup [38], since other antibodies, namely anti-FLE antibodies, display cross-reactivity that may promote antibody-dependent enhancement [32]. Briefly, ADE of infection occurs when cross-reactive antibodies or sub-neutralizing concentrations of antibodies generated in a primary infection facilitate viral entry in a secondary infection by a heterologous serotype or cross-reactive strain [30,39,40,41]. Pathogenesis of viral infection is enhanced through binding of antibodies to Fc receptors expressed on cells of the mononuclear phagocyte system, enabling not only the entry of the virus, but also viral evasion from host antiviral and immune responses [2,30,39]. Moreover, being EDE antibodies binding region on prM-E dimers interaction site (and essential for viral replication being conserved amongst strains), it will have low risk of inducing escape mutations [38]. Given EDE2 antibodies poorer affinity in contact points of the variable 150 loop, these are thus somewhat inferior to EDE1 antibodies [38]. All considered, EDE1 might thus be the preferential option.

#### 4.1.3. ZKA190-10

The mAb ZKA190 was isolated from a panel of anti-ZIKV neutralizing human antibodies [42]. The epitope of ZKA190 was located in the lateral region of domain III of ZIKV E protein, specifically loops BC, DE and FG, and part of the domain I-domain III linker [42]. These residues are conserved in 217 ZIKV strains [42]. Therefore, they may be relevant regions for the development of future antibody vaccines against ZIKV. Surprisingly, ZKA190 also neutralizes Uganda 1947 MR766 strains, which contain substitutions in these residues [42], suggesting the antibody may target other regions of the virus. ZKA190 was shown to neutralize ZIKV strains from Africa, Asia and the Americas, with IC_50_ values in the nanomolar range (0.004 to 0.05 nM). It seems to act at a post-attachment step, likely membrane fusion [42]. Moreover, Wang et al. referred the possibility of an additional neutralization mechanism. The observation of increasing viral amounts on the cell surface associated with increasing antibody concentrations suggests virus inactivation through aggregation, by simultaneously engaging epitopes on different particles of ZIKV, as later confirmed by dynamic light scattering [42]. Pertaining to ADE phenomena, in vivo results indicated that ZKA190 did not elicit ADE, even at doses expected to provide only partial neutralization [42]. Despite in vivo evidence, ZKA190 triggered ADE in vitro [42]. Prophylaxis with ZKA190 protected mice from mortality and morbidity, with survival rates of 80 to 100% (15 mg/kg) and reduction of viral titers, after challenge with ZIKV strain MP1751 (African lineage) [42]. Furthermore, one resistant mutant containing a domain III E370K mutation was identified [42]. The emergence of resistant mutants poses a challenge for antibody-based vaccines, as they can render potential therapeutics obsolete. To minimize escape mutations risk, Wang et al. combined the potently neutralizing ZKA190 with the mAb ZKA185, creating the bispecific antibody FIT-1 [42]. ZKA185 was chosen as it cross-neutralizes ZIKV strains and does not compete with ZKA190, because it targets a different epitope, in domain II of the E protein [42]. FIT-1 preserved the parental antibodies neutralizing potency against ZIKV strains and similar IC_50_ values. Moreover, FIT-1 bound E protein with an affinity superior to that of its parental antibodies and no escape mutations were documented, both in vitro and in vivo, even at lower dosages [42]. As FIT-1 did not elicit immune evasion after 8 rounds of serial passages, authors considered it would be an unlikely event, since escape mutations were reported after 3 to 4 passages in studies with ZKA190 and ZKA185 [42]. Virus inhibition by FIT-1 seems to occur by the same mechanisms described for ZKA190 [42]. In addition, FIT-1 demonstrated capacity to block in vitro ADE mediated by prM mAb DV62 and revealed its therapeutic potential in in vivo studies, increasing survival without apparent morbidity and reducing ZIKV viral titers [42].

#### 4.1.4. WNV-86

WNV-86 is a human monoclonal antibody selected from a cluster of 10 mAbs isolated from WNV infected individuals [43]. In vitro, WNV-86 demonstrated effective neutralization of WNV and was shown to neutralize 50% of virus infectivity at 2 ng/mL [43]. According to Goo et al., WNV-86 likely aimed to an epitope located in domain I or II of WNV E protein [43]. Other anti-WNV antibodies have been reported, several of them displaying preferential neutralization of partially mature virions, which still contain prM proteins in their surfaces [43]. Partially mature virions contain structural characteristics of both mature and immature virions, namely the smooth surfaces characteristic of mature virions, plus the prM-E heterotrimeric spikes identified in immature virions [43]. This distinct structure allows exposure of hidden epitopes, which can be better targeted by neutralizing antibodies [43]. However, WNV-86 preferentially targets epitopes displayed on mature virions (that do not have prM), as IC_50_ values required for neutralization of virus particles lacking prM was 4-fold lower than for virus particles containing prM [43]. In vitro selection of escape variants identified a single threonine to asparagine change in residue 64 of E protein domain II, resulting in incorporation of an N-linked glycosylation site, and a second threonine amino acid substitution to lysine in residue 208, also in domain II [43]. Further analysis revealed that WNV particles carrying this second mutation at amino acid residue 208 still displayed neutralization potency and that both mutations were required to inhibit neutralization by WNV-86 [43]. Despite these findings, the precise binding footprint of mAb WNV-86 is still unknown [43]. Besides in vitro evidence, WNV-86 also demonstrated in vivo efficacy and was shown to reduce WNV-infected mice mortality [43]. Furthermore, mice protection was attributed to WNV-86 direct inhibition of virus infection and dissemination, since both wild-type and LALA (a Leu234Ala/Leu235Ala mutation, commonly used to disrupt antibody effector functions) versions of WNV-86 were able to reduce viral titers in the spinal cord and brain of challenged mice [43].

#### 4.1.5. ZIKV-117

ZIKV-117 is a monoclonal antibody isolated from a cluster of mAbs demonstrating affinity for ZIKV E protein [44]. Sapparapu et al. localized the epitope of ZIKV-117 at domain II of E protein, in a region across two adjacent dimers at the dimer-dimer interface [44]. No escape mutants of ZIKV-117 were reported and the mAb demonstrated capacity to neutralize several ZIKV strains, with IC_50_ values ranging from 5 to 25 ng/mL [44]. Neutralized strains included MR 766 and Dakar 41519 (African lineage), Malaysia P6740 and H/PF/2013 (Asian lineage), and Brazil Paraiba 2015 (American lineage) [44]. ADE of disease is one of the main concerns associated with the development of flavivirus antibody-based vaccines [44]. Regardless, ZIKV-117 possesses a restricted type-specific binding pattern and demonstrated not to be cross-reactive with DENV serotypes 1 to 4, as well as WNV E protein [44]. In vivo, ZIKV-117 was shown to protect mice (previously treated with anti-IfnarI mAbs) challenged with the ZIKV African strain Dakar [44]. ZIKV-117 also demonstrated to improve fetal outcome in pregnant mice when administered before ZIKV inoculation at a single dose of 250 µg [44]. Other conducted experiments suggested a possible effect in prevention of vertical transmission of ZIKV, as ZIKV-117 treated pregnant mice displayed lower virus levels in the placenta and reduced viral titers were also found in fetal brain in the progeny of treated mice [44]. ZIKV-117 titers in the placenta and fetal brain were shown to be higher than the IC_50_ neutralization value, an unexpected result since levels of Fc receptor in the mouse placenta tend to be lower than those of other mammalians [44]. Viral RNA levels in dams’ brain and serum were also reduced by ZIKV-117 treatment [44]. The apparent protective role of ZIKV-117 in the pregnancy model was thought to be due to direct neutralization by the mAb, as studies with the LALA version of ZIKV-117 lead to similar results [44]. Additionally, post-exposure efficacy was reported by Sapparapu et al., as administration of ZIKV-117 resulted in a marked reduction of viral burden in dams, in the placenta and in the fetus at embryo day 13.5 [44]. Lastly, pathophysiological analysis reinforced the previous results: decreased placental damage, trophoblast cell death, and increased body size of fetuses was observed in comparison to control-treated dams [44]. Notwithstanding, the possibility of extrapolation of these observations to humans remains unclear, due to significant differences in placental architecture [44].

#### 4.1.6. Other Antibody-Based Approaches

There is still more work in progress that can inspire other antibody-based drugs and/or therapeutic approaches, albeit in a slightly more conceptual phase still. For example, Schenker and Sagiv provided methods for a potential ZIKV infection treatment and/or prophylactic intervention that focus on protecting both fetus and pregnant women against ZIKV infection. The treatment would consist of enriched anti-ZIKV human immunoglobulin preparation, potentially effective against different genotypic variants or strains of ZIKV [45]. This was tested with anti-ZIKV IgGs purified from the plasma of seven convalescent donors, at 92 mg/mL, with complete neutralization of ZIKV in K562 cells [45]. The proposed treatment should be able to prevent cross-reactions with a second species of *Flaviviridae*, due to its neutralizing capacity, reducing the possible occurrence of ADE upon a subsequent infection by another flavivirus species, strain and/or serotype [45]. To determine a therapeutic dose, young immunocompromised mice lacking the receptor for type I interferon were infected with 1 × 10^3^ PFU/mouse by subcutaneous route [45]. According to the natural course of disease, by day 5 post-infection (p.i.) mice began to lose weight, by day 6 p.i. hindlimb weakness was observed, and by day 7 p.i. the weight reduction was about 15–25% of the starting weight and partial to complete paralysis was expected [45]. Different anti-ZIKV antibodies doses were administered via intraperitoneal or IV route at days 1 and 7 p.i., and samples of blood, spleen, liver, brain, and ovary collected for virology and microscopic analysis [45]. To determine whether the treatment is effective in protecting the fetuses, pregnant female mice lacking the receptor for type I interferon were then treated with the previously determined therapeutic dose of anti-ZIKV IgG at embryonic day 5.5 and infected with 1 × 10^3^ PFU/mouse at embryonic day 6.5 [45]. After birth, newborns were evaluated for intrauterine growth restriction, ZIKV infection and injury to the fetal brain [45]. Another proposed method for preclinical studies in a pig model was also presented, consisting of trans-uterus injection of treatment into the amniotic sac, peritoneal cavity and intra-allantoic injection of selected fetuses [45]. At the 14th day after treatment sows and fetuses were sacrificed and tissue samples of fetuses were collected for examination and, if the treatment was successful, prevention of ZIKV transmission from infected fetuses to the adjacent treated fetuses would be expected [45].

Overall, as mentioned above, several promising antibody-based findings have been achieved, both in vivo and in vitro. Moreover, ongoing studies to develop better animal models, may give rise to further improved methodologies. As such, all this paves the way for future antibody-based therapeutic approaches, which can be complemented with other strategies, described hereafter.

### 4.2. Peptide-Based Therapeutic Approaches

Compared to antibody-based approaches, peptide-based developments are somewhat lagging. There are, however, several advances. As before, please refer to the concluding remarks section for a comment on the most advanced developments.

#### 4.2.1. Ri57

Michael et al. proposed short-chain peptides directed against flaviviruses, capable of obstructing key regions of envelope glycoproteins [46]. Ri57 is a peptide with 28 amino acid residues arranged in an enantiopure D-amino acid sequence. Ri57 was shown to display inhibitory activity against DENV serotypes 1 to 4, at 20 µM, with percentages of inhibition of 100% ± 0.0%, 97.8% ± 1.4%, 90.1% ± 8.5%, and 94.4% ± 6.2%, for DENV1, DENV2, DENV3 and DENV4, respectively [46]. The peptide also inhibits ZIKV infection with high inhibitory percentages (71.0% ± 22.5% and 95.8% ± 2.8% for RI57 concentrations of 20 and 35 µM, respectively) [46]. Next, Michael et al. studied Ri57 inhibition mechanism. The peptide’s inhibitory activity was not attributable to cellular toxicity effects, as observed by mitochondrial reductase activity [46]. Instead, Ri57 directly inhibited virus binding to cells [46]. As to its mechanism of action, experimental evidence suggests that Ri57 acts as inhibitor of DENV and ZIKV virus fusion, blocking entry into the host cell and consequently infection [46]. Researchers aimed at obtaining a peptide capable of resisting to peptidase activity [46]. Normal human serum is composed of numerous proteolytic enzymes with capacity to degrade potential therapeutic peptides [46]. Therefore, to be an antiviral candidate with potential in vivo capacity, peptides must be resistant to such enzymes [46]. Ri57 remained completely intact in a solution of peptide in 1:2 dilution of normal human serum, at 37 °C for 24 h, and retained its inhibitory activity against DENV2 when exposed to trypsin [46]. This shows that the peptide has potential to be used in drug development strategies.

#### 4.2.2. Tat-beclin-1

Tat-beclin-1 is an autophagy-inducing peptide, containing HIV-1 Tat protein transduction domain and amino acid residues 267 to 284 of beclin 1, a protein involved in autophagosome formation [47]. In addition, three substitutions (H267E, S279D and Q281E) are included to increase Tat-beclin-1 hydrophilicity and solubility [47]. The rationale behind relates to the known importance of pathways of autophagy in the defense against infection [47]. In fact, mice lacking autophagy genes or with hypomorphic alleles of these genes were more susceptible to lethal viral infections, and genetic knockout or knockdown of such genes led to increased replication of several viral infections [47]. Thus, strategies capable of increasing infected cells autophagy could represent a possible mechanism for prevention and/or treatment of human viral diseases [47]. Levine et al. demonstrated that cells treated with Tat-beclin-1 30 µM, 4 h post-WNV infection, had lower viral titers when compared to control [47]. Tat-beclin-1 also demonstrated to be effective against WNV in vitro, with 10 µM of Tat-beclin-1 resulting in 10 to 50-fold reductions of WNV titers [48]. Reduction of viral titers by Tat-beclin-1 was not due to peptide cytotoxicity, but rather the result of antiviral effects leading to increased autophagy [48]. Additionally, Kawata et al. reported that prophylactic treatment with Tat-beclin-1 demonstrated antiviral activity of this peptide against a variety of positive strand RNA viruses [48]. Besides, in vivo efficacy of Tat-beclin-1 has also been shown, with Tat-beclin-1 D-form improving the clinical outcome in a neonatal mouse model of WNV central nervous system infection, reducing the mortality of WNV-infected mice [48]. Further analysis demonstrated that Tat-beclin-1 treatment let to lower WNV antigen levels in mice brains, lower registers of neuropathology, and less cell death [4]. Taken together, findings seem to support the efficacy of the peptide Tat-beclin-1 both in vitro and in vivo against WNV infection. Noteworthy, in the case of DENV (as well as other viruses, such as hepatitis C and vesicular stomatitis viruses), there is data indicating that activating autophagy may potentiate infection [49,50,51,52,53,54,55]. Moreover, lipids play an important role in viral replication, namely of DENV, with an autophagy-mediated processing of lipid droplets occurring [53,56]. Thus, at least for DENV, autophagy inducing peptides as antivirals must be evaluated very carefully. Hence, a strategy against flaviviruses based on beclin-1 and relying on autophagy may only be of use to WNV infection (if applicable at all).

#### 4.2.3. WLBU-2

WLBU-2 is a 24-residue cationic peptide with antimicrobial activity predicted to result of its interaction with negatively charged lipid membranes, leading to bilayer disruption [57]. A similar antiviral activity has also been suggested against a diversity of enveloped viruses [57]. Mammalian virus membranes do not tend to have negative surface charge, but are richer in cholesterol than host cells, and this higher cholesterol content seems to be needed for viral infectivity [57]. As such, adding a cholesterol recognition amino acid consensus (CRAC) motif to WLBU-2 could direct its activity to cholesterol-rich viral envelopes, prompting virus inactivation [57]. These CRAC modified peptides (LWYIK, LWYIK2 and VWYVK2) were shown to be up to 10-fold more potent antivirals than unmodified WLBU-2 [57]. VWYVK2 was the most active of these molecules, being active even at the lowest concentration tested (0.39 µM), with 77% reduction of plaques [57]. At the same concentration, WLBU-2 reduced viral plaques only by 16% [57]. In addition, IC_50_ values of CRAC-modified peptides were approximately 10-fold lower than for unmodified WLBU-2 [57]. To understand if unmodified WLBU-2 and CRAC modified peptides led to significant cytotoxicity in treated cells, hemolysis and MTS assays were performed [57]. Despite data reporting increased CRAC modified peptides hemolytic activity, when compared to unmodified WLBU-2 (≈3-fold higher), the concentration at which modified peptides induced 50% hemolysis was still higher than that needed to induce 50% viral inactivation [57]. For all CRAC modified peptides, less than 10% hemolysis was observed at concentrations below 0.78 µM [57]. As to MTS assays, it was shown that CRAC modified peptides and WLBU-2 had similar cytotoxicity [57]. Therapeutic levels of CRAC modified peptides were lower than the cytotoxic levels [57]. Researchers also found that adding two CRAC motifs to WLBU2 did not alter activity as compared to peptides with a single CRAC motif, suggesting the absence of an additive effect with multiple CRAC motifs [57]. Furthermore, other mechanisms of inactivation besides lipid disruption were hypothesized. Contrary to expected, DENV (with high protein-to-lipid ratio in its envelope) was the most sensitive to CRAC modified peptides inactivation [57]. Therefore, other mechanisms could be at play, namely viral entry blockage due to these highly cationic peptides interaction with negatively charged cellular receptors of DENV, leading to inhibition of dynamics between viruses and their receptors on the cell surface [57]. Overall, this supports further studies of potential peptide-based drug development approaches.

### 4.3. Other Therapeutic Approaches

The type I interferon (IFN) family is a multi-gene cytokine family encoding 13 partially homologous IFNα subtypes in humans [58]. IFNα subtypes are recognized for inducing an antiviral state in both virus-infected and uninfected cells, doing so by inducing a program of gene transcription that interferes with various stages of the viral replication cycle [58]. Furthermore, studies with type I IFN receptor (IFNAR1)-deficient mice provided evidence of the protective role of IFNα against viruses in vivo [58]. This property of IFNα was also reinforced by studies in which exogenous IFN was used to treat viral infections [58]. In fact, most viruses devote part of their limited genome to mechanisms that perturb IFNα/β production and/or IFNα/β-mediated signaling, inhibiting the induction of IFN-stimulated genes [58]. This alone demonstrates these cytokines importance in protecting against viral infection [58]. Examples of prototypic viruses that benefit from inactivation of IFNα include flaviviruses such as WNV, alongside avian Influenza, SARS-CoV-1 and smallpox viruses, among other [59].

Ampligen is a synthetic double stranded ribonucleic acid (dsRNA) molecule, containing a rugged structure that increases its resistance to molecular unfolding, and acts as a selective Toll-like receptor 3 agonist [60]. Binding of dsRNA to Toll-like receptor 3 induces expression of α and β interferons, and cytokine production, leading to an antiviral state within various cells [61]. Thus, Ampligen acts as an interferon-inducing molecule. Its efficacy has been demonstrated against both flavivirus and alphavirus-associated encephalitis in experimental animal models [62]. This molecule could serve as a WNV prophylactic treatment: it has been administered (intraperitoneal injection) to mice exposed to WNV (at a dose of 13 mg/kg), preventing mortality in mice [62]. Ampligen administration was shown to reduce viral titers to levels below the detection limits, supporting its drug efficacy [62]. Notwithstanding, Ampligen prophylactic treatment (4 to 8 h pre-infection) did not result in statistically improved survival [62]. Moreover, in a separate experiment, Ampligen administered 4 to 6 h before viral challenge was shown to display no statistical difference compared to saline control [62]. Despite that, Ampligen treatment was associated with improved weight change [62]. Thus, it was efficacious in vivo only when treatment began at least one day before WNV exposure [62]. As other antiviral gene modulation strategies resorting interferon have been reported, this may become another tool in the interferon-based prophylactic/therapeutic arsenal, although likely not fully effective on its own.

Strategies employing alternative targets are currently under development. Namely, approaches directed against the structural C protein of flaviviruses, more precisely through blockage of interactions between the latter and host and/or viral elements, as reviewed elsewhere [4]. As an example, Martins et al. have developed pep14-23 [29,55,56], a promising drug lead, which seems to inhibit the interaction of DENV C protein with host intracellular lipid droplets (LDs) in in vitro studies [29,30]. This is an essential interaction for viral replication [30]. Lipids and lipid droplets are in fact quite important for DENV infection [28,29,53,56]. Thus, approaches such as that of pep14-23 may be of particular interest in the development of anti-flavivirus drugs [4,28,29,54,55,63,64].

Summing up, a list of potential therapeutic approaches is presented below (Table 2).

## 5. Concluding Remarks

The continued expansion of vectors beyond previously known endemic regions and the establishment of competent mosquito species in regions of Europe have raised the awareness of the potential risk of flavivirus infections. Increase in the frequency of outbreaks and the emergence of cases in regions with a more temperate climate have highlighted flaviviruses’ changing epidemiology and ability to successfully adapt to new contexts. Such concerns regarding flavivirus infections are no longer circumscribed to the scientific community, as proven by increasing implementation of surveillance programs in several countries and territories. Regardless of the overall mortality rates associated with these infections, the economic burden is substantial and especially harmful to socioeconomically disadvantaged regions [1,2,3,4,65]. Although slow, progress towards the development of potential therapeutics is ongoing. As described herein, several antiviral agents directed against DENV, WNV and ZIKV are currently under investigation. Antibody-based compounds have yielded the most promising results, with demonstration of both in vitro and in vivo efficacy. In our view, antibody-based therapies are presently the most advanced and promising therapeutics, especially those based on monoclonal antibodies against specific domains of the E protein. This suggests that this structural protein is indeed a good target of interest for the development of future antiviral drug therapies. Further studies are still necessary, but the three mentioned monoclonal antibodies (FIT-1, WNV-86 and ZIKV-117) decreased mice mortality and one of them (ZIKV-117) improved the outcomes in the progeny of pregnant mice facing ZIKV challenge. However, the use of antibody-based vaccines and/or treatments faces some challenges, such as cross-reactivity amongst epitopes of different flaviviruses, leading to ADE. Although, given the divergent evidences regarding the in vivo demonstration of ADE in mouse models, it is not yet certain and may depend on the flavivirus under assessment [56]. Additionally, virion proteins can be also targeted by peptide-based compounds, disrupting crucial steps of the viral life cycle (e.g., Ri57). Other compounds with alternative mechanisms of action have also been explored, including peptides inducing autophagy of infected cells (e.g., Tat-beclin-1) and modulators of interferon expression (e.g., Ampligen), with in vivo activity against WNV. Reports of the efficacy of such heterogeneous approaches reflect the multiple potential targets encoded by flaviviruses, as well as the potential of host-directed antivirals. As a whole, the compounds presented demonstrate that several treatments based on a variety of approaches may become feasible options in the near future. Nevertheless, more research regarding efficacy and safety is needed for the development of a potential antiviral therapy. Strategies employing alternative targets are currently under development, for instance, approaches directed against the structural C protein of flaviviruses, more precisely through the blocking of interactions between the latter and host and/or viral elements, as reviewed elsewhere [4]. As an example, we developed pep14-23 [29,55], a promising drug lead, which inhibited the interaction of DENV C protein with host intracellular lipid droplets (LDs) in in vitro studies [29]. This is an essential interaction for viral replication [30], thus, approaches such as that of pep14-23 may be of particular interest in the development of new anti-flavivirus drugs [4,29,54,55].

To conclude, along with continued investigation efforts, implementation of vector control strategies and other countermeasures that limit emergence and re-emergence of flavivirus outbreaks are also crucial to lessen the burden caused by their infection. Thus, a combined multifactorial approach is the best to follow, especially given flaviviruses adaptability and the vector role in epidemics. Multi-pronged policy planning strategies are more likely to yield good results, relying both on preventive actions (vaccine-based, whenever possible, plus vector control), as well as on therapeutics (post-infection treatments, possibly deriving from the potential antivirals discussed above). Such strategies are thus recommended, alongside additional research on this topic, aiming at future effective specific antiviral treatments.

## Figures and Tables

**Table 1:** Flavivirus infections outcomes, including severe symptoms that may lead to fatalities.

	DENV Infection	WNV Infection	ZIKV Infection
*Mild symptoms*	Flu-like syndrome	Flu-like syndrome	Flu-like syndrome
	Retro-orbital pain	Nausea and vomiting	Conjunctivitis
	Nausea and vomiting		
	Rash		
*Severe symptoms*	Shock Respiratory distress Severe bleeding Organ impairment	Neuroinvasive Disease	Guillain-Barré syndrome
		Encephalitis Meningitis	Specific fetal syndrome
			Microcephaly
			Other congenital malformations

**Table 2:** Summary of key characteristics of the compounds presented.

Type	Compound	Mode of Action	Evidence	Stage	Ref.
**Antibody-Based**	AC-10	Likely inhibits viral binding and/or membrane fusion by targeting epitopes in the lateral ridge of domain III and domain I of E protein	In vitro against ZIKV	Pre-clinical	[36]
	EDE1	Targets epitopes in the E protein	In vitro against DENV1-4 and ZIKV	Pre-clinical	[37]
	FIT-1	Inhibition of a post-attachment step (likely fusion) by targeting an epitope in the lateral ridge of domain III of ZIKV E protein	In vitro and in vivo against ZIKV	Pre-clinical	[41]
	WNV-86	Most likely targets an epitope in domain I or domain II of E protein. Preferentially recognizes epitopes of mature virions.	In vitro and in vivo against WNV	Pre-clinical	[42]
	ZIKV-117	Targets an epitope on domain II of E protein	In vitro and in vivo against ZIKV	Pre-clinical	[43]
**Peptide-Based**	Ri57	Inhibits viral fusion by targeting regions of E protein	In vitro against DENV1-4 and ZIKV	Pre-clinical	[45]
	Tat-beclin-1	Induction of autophagy	In vitro and in vivo against WNV	Pre-clinical	[46,47]
	WLBU-2 modified peptides	Inhibition through interaction with viral membranes	In vitro against DENV	Discovery	[48]
**Other**	Ampligen	Induction of interferon expression	In vitro and in vivo against flaviviruses	Pre-clinical	[51,53]

## Data Availability

Not applicable.
